# Behind the times: a brief history of motivation discourse in problem-based learning

**DOI:** 10.1007/s10459-019-09923-3

**Published:** 2019-10-14

**Authors:** Lisette Wijnia, Virginie F. C. Servant-Miklos

**Affiliations:** 1 Erasmus University College, Erasmus University Rotterdam, Rotterdam, The Netherlands; 2 Roosevelt Center for Excellence in Education, HZ University of Applied Sciences, Edisonweg 4, 4382 NW Vlissingen, The Netherlands; 3 Department of Planning, Aalborg University, Ålborg, Denmark

**Keywords:** Problem-based learning, Motivation theory, Achievement-goal theory, Self-determination theory

## Abstract

That idea that problem-based learning (PBL) is more motivating that traditional education has been prevalent since the inception of PBL at McMaster University in the late 1960s. Evidencing this through empirical research, however, has proven to be a lot more problematic. This paper retraces how the discourse on motivation started from a laymen’s conception in the early days of PBL, and slowly evolved into a field of scientific inquiry in the 1980s and 1990s. However, looking at the evolution of motivation theory over the same period, we show that motivation discourse in the burgeoning literature on motivation and PBL remained largely wedded to the laymen’s approach, and failed to catch up with the new achievement-goal theory and self-determination theory approaches. This paper proceeds to analyse the explosion of studies on PBL and motivation after 2000, acknowledging efforts to move away from anecdotal accounts and provide theoretical grounding to the research. However, once again, we show that the majority of the research employed outdated motivational measures that do not fully grasp the complexity of contemporary motivation theory. The paper concludes on the observation that single-course and curriculum-wide research interventions have yielded no conclusive results on the effect of PBL on intrinsic motivation, and that future research should therefore seek to use up-to-date motivational constructs in more targeted interventions.

## Introduction

Motivation is one of the psychological aspects of problem-based learning (PBL) that has received the most research attention. In the 50 years since PBL’s inception at McMaster University, there have been over 1000 scientific papers published on the link between PBL and motivation. This makes sense to educators familiar with PBL. The learning format in which a small group of students tackles a realistic problem that acts as the starting point for learning under the guidance of a tutor (Barrows and Tamblyn 1980), seems inherently more motivating than sitting through long lectures. It was this very intuition that prompted John Evans and his colleagues Bill Spaulding, Fraser Mustard, Jim Andersen, and Bill Walsh to develop PBL in the first place, at a time when medical education was a tedious affair that involved sitting through years of basic sciences before a student could see a patient (Servant 2016). Moving on from this intuitive starting point in the 1970s, education scholars and practitioners since the late 1980s have tried to find empirical support that PBL can indeed generate better motivational outcomes than traditional educational approaches and through which mechanisms this effect is achieved. Finding such empirical support, they hoped, would help to convince other schools and practitioners of the effectiveness of PBL. Difficulties immediately surfaced as PBL educators struggled to translate the layman’s conception “motivation” into a metric that could be used to improve the curriculum (Berkson 1993; De Volder et al. 1986). Furthermore, the growing body of research on PBL curricula failed to find empirical support for the claim that PBL can enhance student motivation, and reported on motivational problems that occur during group meetings (Dolmans and Schmidt 2006; Galand et al. 2010; Wijnen et al. 2018; Wijnia et al. 2011).

This paper hopes to show that such disappointments are primarily caused by misunderstandings of motivation theory and the psychological science that surrounds it—misunderstandings that cause, for instance, educators to conflate autonomy and independence when the two are not identical (Chirkov et al. 2003; Ryan and Deci 2006). In the following sections, we will retrace the historical origins of motivational discourse in PBL, particularly at McMaster University and Maastricht University, the first two universities to offer PBL programmes. Then, we will look at the evolution of motivation discourse in the literature on PBL, from the 1980s until the 2000s. We will show that although motivation theory has made strides in the past few decades, PBL scholars often seem to be behind the times, applying outdated theories or measures or failing to incorporate all aspects of motivational theory to their programmes and educational experiments. This paper will therefore end on a call for educational scholars interested in motivational aspects of PBL to link in better with the contemporary science of motivation and perform more targeted rather than curriculum-level research interventions.

## The origins of motivation discourse in PBL

### McMaster’s Laymen approach to motivation

Spaulding mentioned student motivation as one of the reasons behind the new problem-based approach at McMaster in a memorandum that defined the first PBL curriculum (Spaulding 1968). This passing mention made no reference to any theoretical understandings of motivation. The first significant mention of motivation as a key driver for PBL came from Dave Sackett, one of the first members of McMaster’s education committee, who wrote in a provisional curriculum proposal from 1968. He labelled medical education as “frequently irrelevant, usually boring and almost without exception detested by its consumers. If this situation is to be any different at McMaster University, an initial premise must assume that students are motivated and that the problem lies in determining areas of student motivation and capitalising upon them” (Sackett 1968).

As the education committee (EC) responsible for developing the curriculum grew and preparations for the opening of the Faculty of Medicine in September 1969 were in full swing, references to motivation popped up time and again in minutes of the EC and memoranda submitted by its members. The most commonly expressed sentiment was that motivation to study was inherent in students, and that a successful study programme should seek to enhance it through contact with patient problems rather than stifle it with boring lectures and disconnected knowledge. This belief was reflected in McMaster’s admissions policy that stated that “the student should select courses according to his interest and motivations” (Ad Hoc Committee on Undergraduate Education 1969). It was also present in the delivery of the programme, with the expressed hope that PBL would enable students to “see the relevance of what they are learning in their future careers. This hopefully maintains a high degree of motivation” (Ad Hoc Committee on Undergraduate Education 1969). It also featured in McMaster’s assessment policies, which were designed to “emphasize the assessment of personal and motivational characteristics rather than purely academic grades in the selection of our students” (Adsett et al. 1969). This commitment to enhancing student motivation through PBL made its way into the literature for the first time when Spaulding (1969) published an article in which he expressed his hope that students would maintain a “high degree of motivation” through their studies (p. 659).

After the initial flurry of enthusiasm, no doubt buoyed by the Founding Dean’s rejection of his own dull medical school experience (McAuley 1979), talk of student motivation progressively disappeared from the education committee throughout the 1970s, was never formalized into a researchable or instrumental component of PBL, and did not arise as a subject in Neufeld and Barrows’ (1974) seminal article on PBL. The latter focused instead on lifelong learning, self-directed learning, and clinical reasoning skills. A short study by Barrows and Tamblyn produced in (1976) did mention increased motivation as an outcome of PBL using simulated patients, on the basis that students in their experimental group sought out more clinical experiences after seeing the simulated patients, but the authors did not make connections to motivational theory. Finally, Barrows and Tamblyn’s 1980 book makes almost no mention of motivation.

### Maastricht’s proto-theoretical approach

When PBL was transferred to Maastricht University in 1974, this came with the addition of an Educational Research and Development Centre (ERDC) nominally headed by Wijnand Wijnen, but de facto run by Henk Schmidt and Peter Bouhuijs (Servant 2016). Mentions of student motivation in various minutes of meetings and memoranda around the time of the opening Maastricht’s Faculty of Medicine were initially not much more developed than McMaster’s. However, the existence of the ERDC opened the door to a formal investigation of the link between student motivation and PBL. In 1977, Schmidt (1977) developed a research programme, which explicitly mentioned motivation for study as an area of interest. While acknowledging that research of a general nature had been done of the subject, he stated: “the implementation of this [research] in a more concrete situation and the results of this within a medical education programme asks for more depth, breadth and research” (Schmidt 1977). Shortly after, a tutor training manual *Het Tutorensysteem* (Bouhuijs et al. 1977) edited by Bouhuijs, offered the first, albeit brief cognitive psychological discussion of motivation in PBL. They proposed that studying an unknown problem would be “a strong motivating factor for students to fill the discrepancy between actual and required knowledge and skills through intensive study” (p. 3). This framing reflects Schmidt and Bouhuijs’ embracing of constructivist psychology in the late 1970s (Servant-Miklos 2019). In the early 1980s, Schmidt formalized his work on motivation, looking specifically at *intrinsic* motivation as part of his Ph.D. thesis (Schmidt 1982). Schmidt used the concept of epistemic curiosity developed by the behaviourist psychologist Daniel Berlyne in the 1950s (see Berlyne 1954, 1978) to explain how students confronted with a problem-induced conceptual conflict would try to reduce cognitive uncertainty. He set up two experiments in which he first measured the extent to which problem analysis in PBL promotes intrinsic motivation, and then tested the relationship between intrinsic motivation and study performance (Schmidt 1983). The first experiment showed that people who had undergone the problem analysis process (initial discussion phase) reported a higher interest in reading more about the topic afterwards. The second experiment, however, showed no significant relationship between intrinsic motivation and study results. This led Schmidt to hypothesize that intrinsic motivation was a situational phenomenon, rather than a long-term process. These studies opened the door to the theorization of motivation in PBL through the intrinsic motivation approach.

### The turning point in motivation theory and its (lack of) impact on PBL

**Fig. 1:**
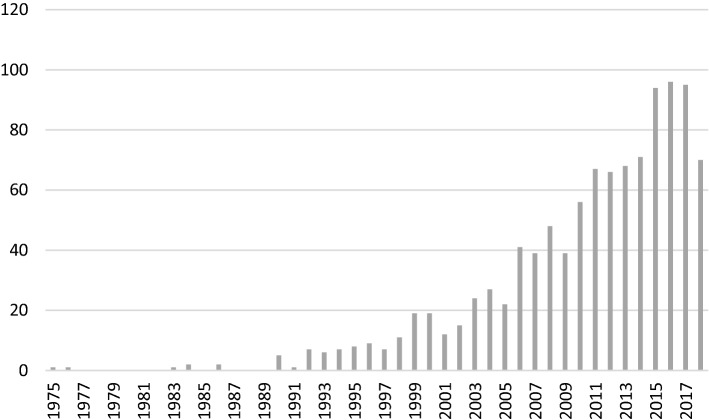
Number of unique papers identified through database search using the search terms “problem based learning” with “motivation” or “situational interest”. Articles from 2019 (n = 21) were excluded from the figure

From the late 1980s, the volume of publications on the subject of motivation in PBL expanded significantly. In order to examine the developments of motivation discourse within that explosion of literature, we switched from the historical approach used in the previous section to a database search using the search terms “problem based learning” AND (“motivation” OR “situational interest”) in Web of Science Core Collection and Medline, PsycINFO (Ovid interface), ProQuest Dissertations and Theses, ERIC, and Social Science Database (ProQuest platform). Our search produced 1077 documents after the removal of duplicates (Date searched: May 17, 2019). What our search shows is that, as evidenced in Fig. 1, after the initial take-off in the 1990s, the discourse on motivation became more popular in the literature on PBL from the turn of the millennium, onwards.

Further complicating the research and discussion of the topic of motivation and PBL, is the increasing complexity of motivation as a complex subject of inquiry, which has spawned many different conceptualizations, theories, and measures over the years (e.g. Murphy and Alexander 2000; Graham and Weiner 2012).

### The development new theories of motivation

The curiosity drive stance utilized by Berlyne (1954) did not survive the all-out assault on behaviourism launched by the cognitive revolution that started in 1956 (Miller 2003). First to emerge from the rubble was Deci (1971), whose work on the undermining role of external rewards on intrinsic motivation formed the starting point of self-determination theory (SDT; Deci and Ryan 1985, 2000; Ryan and Deci 2000, 2017). Its basic premise was that all humans experience the need for autonomy, competence, and relatedness (basic needs), and these can be either satisfied or thwarted. Motivation, defined as the reasons for undertaking an activity, exists on a self-determination continuum from amotivation to intrinsic motivation, with various stages of extrinsic motivation in between, and where a person sits on that continuum depends on the extent to which their needs are satisfied (Ryan and Deci 2000). As such, the motivational descriptors in the SDT continuum emphasize the quality, rather than the quantity of motivation experienced (Deci and Ryan 2008). Moving away from the classical distinction between intrinsic and extrinsic motivation, SDT distinguished between autonomously motivated students and students experiencing controlled motivation. The former experienced volition whilst working on a task and either did so out of interest (intrinsic motivation) or for self-improvement purposes (identified motivation); while the latter experienced internal feelings of shame or guilt (introjected motivation) or social pressure (external motivation). For the purposes of PBL research, it is important to note that Ryan and Deci (2006) did not equate autonomy with independence; autonomy meaning to act with a sense of choice, and independence meaning to function alone.

The second major approach to motivation that emerged in the 1980s was the Achievement Goal Theory (AGT). Unlike SDT, it does not have clear founding fathers, but is related to prior work on mastery goals (developing competence) and performance goals (outperforming others) by scholars such as Ames and Archer (1988), Dweck (1986) and Nicholls (1984; see Senko et al. 2011). The received wisdom of the time was that mastery goals were associated with positive outcomes (interest, persistence, deep learning, positive emotions), but often unrelated to achievement, whilst performance goals yielded mixed results on achievement. This distinction was refined by Elliot (1999) and Pintrich (2000) who distinguished between performance-approach (striving to outperform others or appear talented) and performance-avoidance goals (striving to avoid doing worse than others or appearing less talented), a demarcation that was empirically validated (e.g. Elliot and Church 1997; Elliot and Harackiewicz 1996). Elliot and McGregor (2001) argued the approach/avoidance distinction can also be applied to mastery goals, resulting in four goals that form the basis of AGT: mastery-approach (striving to learn or improve skills), mastery-avoidance (striving to avoid learning failures or skill decline), performance-approach (striving to outperform others or appear talented), and performance-avoidance (striving to avoid doing worse than others or appearing less talented) goals.

Because AGT was developed by several theorists coming from different theoretical frameworks, AGT is marred by different labels, measures, and definitions that yield unresolved controversy amongst proponents of the theory (Elliot et al. 2011; Hulleman et al. 2010; Senko et al. 2011). One of the controversies is the mastery versus multiple goals debate. Whereas some scholars believe that only mastery goals lead to optimal learning outcomes, others believe performance goals can have beneficial effects as well. Another issue concerns the definition and measurement of performance goals where some scholars emphasize the “outperforming others” dimension and others focus on “demonstrating ability”. Hulleman et al. (2010) showed that these two dimensions are differently associated with performance outcomes.

Aside from SDT and AGT, Atkinson’s Theory of Achievement Motivation (1957), leading to the Expectancy-Value approach (Graham and Weiner 1996) was popular from the 1960s up to 1980. The theory posited that what is undertaken depends on the perceived likelihood that the behavior will lead to a goal (expectancy), and the subjective value of that goal. The influence of this approach waned in the 1980s but it was popular enough to crop up in a lot of PBL research, as we shall see. Furthermore, expectancy and value are key components in a popular motivation survey, Motivated Strategies for Learning Questionnaire (MSLQ; Duncan and McKeachie 2005; Pintrich et al. 1991) that has been used to evaluate the effectiveness of PBL in a significant number of studies.

### PBL literature’s theoretical lag

Despite the promising developments in motivation theory in the 1980s and the growing body of research on PBL, the bulk of the articles written on motivation and PBL in the period between 1980 and 2000 continued to use a layman’s conception of motivation. This resulted in the term “motivation” being bandied about without further definition in the majority of the articles written in that period (e.g. Barrows 1986; Birch 1986; Happell 1998; Ostwald et al. 1992; Philp and Camp 1990; Sobral 1995; Thomas 1997) and used alongside anecdotal evidence to justify a change from traditional medical education to PBL (e.g. Gerike et al. 1999; Lucas 1998; Myers et al. 1993; Von Döbeln 1996). Some studies merely used end-of-course evaluations as an indication of the motivational effects of PBL (Alabi et al. 1996; Happell 1998; Luthra and Das 1998). A minority of papers attempted to provide empirical evidence for the impact of PBL on study motivation. Building on the empirical studies of Schmidt (1983), De Volder et al. (1986) used the theoretical lens of intrinsic motivation, tied to the ageing paradigm of epistemic curiosity (Johnson and Johnson 1979), rather than the newer theories of motivation presented above. Other empirical papers written over the 1980–2000 period used Bruner’s discovery learning as a theoretical framework (Norman and Schmidt 1992; Win 1990), expectancy-value theory (Bridges 1992), or no theoretical framework at all (Lohse Bayard 1994). Only two studies attempted to understand the link between PBL and motivation through work from AGT or SDT scholars (Eitel and Steiner 1999; MacKinnon 1999). This lag had important consequences on the practice of PBL over the period.

### Impact on the practice of PBL

The problem with the approach taken by the majority of the papers we reviewed thus far is that they put forward the presumed positive effects of PBL on motivation, due to the relevance and meaningfulness of problem scenarios to students, to argue in favour of implementing PBL in medical schools (Barrows 1986; Birch 1986; Ostwald et al. 1992; Philp and Camp 1990). Unfortunately, none of the studies done up until 2000 were able to convincingly conclude that PBL had any significant impact on motivation (Berkson 1993; Thomas 1997), thereby invalidating one of the key arguments of proponents of PBL who have put forward that increasing intrinsic motivation is an important education goal of PBL (Barrows 1986; Norman and Schmidt 1992). In one study, students even reported that the main difficulty that they experienced during a PBL module was lack of motivation (Pau et al. 1999)! Adding to this issue, the 1980s also saw the rise of conflicting conceptions of the purpose of PBL, where a group of medical educators led by Barrows viewed it as a method to hone problem-solving skills rather than as an approach for knowledge acquisition. This was in contravention to the latest developments in cognitive theories of learning, and led to the implementation of ineffectual PBL programmes (Servant-Miklos 2019). The combination of the two factors hindered the development of theoretical support for PBL in the 1990s, and led to a wide diversity of practice variations of PBL. Starting with Harvard University (Moore et al. 1994), medical schools began developing so-called “Hybrid” PBL programmes that ran problem scenarios in small groups alongside traditional education. Though the development of PBL Hybrids is more fundamentally connected with the prevalence of the problem-solving skills discourse (Servant-Miklos 2019), it can also be interpreted as a reaction to the lack of evidence for PBL as a motivating pedagogy for students. Educators may have wondered why, if PBL doesn’t yield greater motivation, they should go through the difficult process of implementing it fully. They believed PBL’s main purpose is to teach problem-solving skills and it was assumed by the managers of Hybrid programmes that this could be done by interspersing problem scenarios in a regular programme.

### Bridging the disconnect between motivation theory and PBL

After the turn of the millennium, the number of papers on PBL and motivation truly exploded, as shown in Fig. 1, and many of these papers comprised empirical studies. The growth in studies reflects both the increasing popularity of PBL as a pedagogical method, and the increasing interest in studying PBL through a scientific lens. The late 1990s and early 2000s also witnessed a particularly impressive growth of PBL in Asia (Servant 2013), and in Science, Technology, Engineering, and Mathematics (STEM) disciplines all over the world (Potvin and Hasni 2014).

### Empirical research still lagging behind theoretical developments

The fact remains that despite the widespread adoption of PBL in medical education and its expansion in other fields, the majority of papers about PBL are produced by educators whose primary research specialty, usually in a sub-field of medicine or engineering, lies outside of the field of education. Education is something they primarily *do*, not something they *study*, with notable exceptions. And yet, the confusion between experiential knowledge gained as an educator and scientific knowledge about education has hampered the field of education studies from the outset, leading some to treat education research as “rocket science” in which complex social phenomena are reduced to “generalizable simplicity” (Regehr 2010), and others as throwing “paper darts” that inevitably crash against the complexity of the phenomena under scrutiny (Norman and Schmidt 2000).

In the light of this observation, it is plausible to link the rise in studies done on PBL and motivation to the availability of the MSLQ (Pintrich et al. 1991) mentioned above, given that it is a convenient, validated questionnaire that offers a wide range of angles from which to measure motivation for medical, STEM, or other educators who wish to study PBL but have no background in motivation theory. Indeed, we see that the MSLQ forms the basis of several empirical studies done after 2000 (Ateş 2009; Liu 2005; Massa et al. 2012; Massa et al. 2014; Sungur and Tekkaya 2006; Vazquez 2008).

A recent meta-analysis of the effectiveness of PBL on motivation looked at all of the studies done over the period using a pre-post and/or independent groups design in which PBL was compared to a teacher-centered/lecture-based group (Wijnia et al. 2019). In the 113 subsamples that were included in the meta-analysis, they found reported effects for various constructs linked to expectancy-value theory, such as students’ perceptions about their own ability (e.g. self-efficacy beliefs) and control over their learning process, investigated in 62 studies (*d *= 0.403), and perceptions of task value (including interest), investigated in 31 studies (*d* = 0.587). Furthermore, an effect was found on students’ attitude toward learning, investigated in 23 studies (*d* = 0.240). Attitude is a popular construct that is often measured in STEM disciplines to indicate a students’ global positive or negative feeling toward learning or engaging in an activity (Germann 1988). However, only 20 studies investigated constructs linked to students’ (intrinsic or extrinsic) goals and reasons for studying as measured in the more recent AGT and SDT approaches to motivation (Wijnia et al. 2019). This is surprising in light of the popularity of these theories in contemporary educational research and the fact that Barrows (1986) and Norman and Schmidt (1992) have argued that one of the goals of PBL is to enhance students’ intrinsic motivation for studying. Of those 20 studies, 13 used the classical distinction between intrinsic versus extrinsic motivation, often using the scales provided by the MSLQ (Pintrich et al. 1991; Wijnia et al. 2019). Only seven studies used actual AGT and/or SDT measures. However, the four studies investigating motivation from an SDT perspective only used autonomous and controlled composites or a relative autonomy score, without differentiating between the subtypes of motivation. Of the four studies that included AGT measures, only two distinguished between performance-approach and performance-avoidance goals and only one differentiated between mastery-approach and mastery-avoidance goals. Combined, the 20 studies showed a near-zero effect (*d* = 0.022), indicating that PBL does not seem to affect students’ intrinsic or extrinsic motives for studying (Wijnia et al. 2019). So in summary, despite the surge in interest in investigating the link between PBL and motivation, after over a hundred empirical studies, we still have very little insight into the effects of PBL on students’ motivation using contemporary motivation theory. The unclear results emerging from the studies that *do* use more updated motivational constructs teach us that the link between PBL and motivation is more complex than the founding fathers of PBL and later PBL practitioners assumed. This tells us that if we mean to improve PBL to trigger motivation in students, more and better targeted research will have to be performed, otherwise we risk seeing more “paper darts”.

### Learning from research on situational interest

One promising avenue of research is the Knowledge-Deprivation Hypothesis of Situational Interest, currently investigated by Rotgans and Schmidt (2011b, 2014, 2019; Schmidt et al. 2011). Situational interest is an emotional state that is triggered by specific aspects of the task or learning environment. It can eventually develop into individual interest, which is a relatively stable and enduring orientation toward a task or domain, through the interaction between the individual and their environment (Hidi and Renniger 2006). Interest and autonomous motivation are closely related, but distinct constructs, in which motivation is the more cognitive and interest is the more affective in nature (Hidi 2006). Using a micro-analytical approach, Rotgans and Schmidt (2011b) measured situational interest before and after students received a problem, immediately after the initial discussion of the problem, after the self-study phase, and after the reporting phase. What they showed was that situational interest increased after the problem was first presented. However, as students acquired more knowledge about the problem, their situational interest decreased. The development of situational interest during the course of a PBL session therefore followed a U-shape. This phenomenon, known as the *knowledge*-*deprivation hypothesis* (Rotgans and Schmidt 2014, 2019), consists of four elements: (1) problem confrontation (retrieval attempt from long-term memory to make sense of the problem), (2) knowledge-retrieval failure (becoming aware of the knowledge deficit or gap), (3) situational interest arousal (awareness of gap and willingness to engage in knowledge-seeking behavior to close the gap), and (4) reduction of situational interest (knowledge acquisition and closing of the gap reduces situational interest). What we learn from this body of research on PBL and interest, methodologically speaking, is that targeting the research to specific elements and moments of PBL can break down some of the complexity of the psychological aspects of PBL. This is a point that should be transposed into research on PBL and motivation.

### Breaking it down

There have been some studies within the past 20 years, especially from the Netherlands, that have attempted to do just that. Studies such as Gijselaers and Schmidt (1990) and Van Berkel and Schmidt (2000) used programme evaluations to investigate how specific elements of PBL influenced time spent on self-study, interest in the subject matter, and achievement. The studies showed that prior knowledge, the quality of group functioning, and problem quality were predictive of perceptions of interest in the subject matter. Sorbal (1994) investigated the role of peer-tutoring on perceptions of meaningfulness and interestingness of the learning process. Other studies have examined the role of tutors’ instructional styles or characteristics such as subject-matter expertise on students’ autonomous motivation or situational interest (Rotgans and Schmidt 2011a; Wijnia et al. 2014), or the role of having a free choice of literature resources to study (Wijnia et al. 2015). Noordzij and Wijnia (accepted) examined effects of problem quality on autonomous motivation and found that problems that are perceived as familiar, problems that result in the intended learning issues, and problems that promote critical reasoning, increase the level of interest in the problem and can in turn affect autonomous motivation.

What these studies show is that by using SDT and/or AGT constructs, refined to take into account the nuances offered by the later developments in the theories, and applied to specific elements of PBL rather than curriculum-level interventions, we begin to piece together the mosaic that is the link between PBL and motivation. It helps to take concrete examples that many PBL educators will be familiar with: it’s a common experience that within the same group, with the same students and same tutor, one high quality problem will be motivating, while a poor quality one will not (Noordzij and Wijnia accepted). As is the experience that, for instance, rigidly following the seven-step approach to PBL (Wijnia et al. 2011) leads to increasing frustration and corner-cutting within PBL groups, and ultimately to the “erosion” of the PBL method (Moust et al. 2005). In that sense, because PBL is actually a combination of so many factors rather than one monolithic approach, even the question of the link between PBL and motivation is a misnomer that hides the complexity of the issue. It is urgent for the discourse on motivation in PBL to catch up with this complexity.

## Conclusion

Although the founders of PBL believed intuitively that working with problems would be more motivating for students than cramming in basic sciences knowledge delivered through lectures for years before seeing a patient, they had no scientific basis for making this assumption. The promise that PBL would be more intrinsically motivating than traditional educational approaches failed to stand up to scrutiny when looked at globally. However, beyond the prevalence of a laymen’s conception of motivation in the early literature, we have shown that the outdated measures employed in the majority of studies and the lack of nuances in studies that make use of contemporary theories could explain this result. PBL is a broad learning approach that combines many interacting elements, namely problems, tutors, group dynamics etc. Each of these individual elements can be of low or high quality, as can the manner in which they are arranged together. And all of these complex factors together can have an effect on students’ motivation. While motivation theory has increasingly endeavoured to reflect these complexities, this has not translated into the research on PBL which has tried and failed to capture motivation as a product of a sum-total PBL environment, rather than a combination of factors within PBL. To design motivating PBL environments will therefore require more focused research on the specific elements within PBL, with the understanding that whereas one specific combination of elements might yield overall greater motivation, another might not, even though both claim to take place in a PBL environment. Such research will not only benefit students, but it will also help the community of PBL scholars and practitioners focus somewhat more the definition of PBL and the elements that make it effective.
